# Unique hemoglobin dynamics in female Tibetan highlanders

**DOI:** 10.1186/s41182-020-00289-6

**Published:** 2021-01-04

**Authors:** Hiroaki Arima, Masayuki Nakano, Sweta Koirala, Hiromu Ito, Basu Dev Pandey, Kishor Pandey, Takayuki Wada, Taro Yamamoto

**Affiliations:** 1 Department of International Health and Medical Anthropology, Institute of Tropical Medicine, Nagasaki University, 1-12-4 Sakamoto, Nagasaki, 852-8523 Japan; 2 Graduate School of Biomedical Sciences, Nagasaki University, Nagasaki, Japan; 3 Department of Nutrition, Faculty of Health Sciences, Kochi Gakuen University, Kochi, Japan; 4 Nepal Development Society, Kathmandu, Nepal; 5 Everest International Clinic and Research Center, Kathmandu, Nepal; 6 Sukraraj Tropical and Infectious Disease Hospital, Kathmandu, Nepal; 7 Nepal Academy of Science and Technology, Lalitpur, Nepal; 8 Graduate School of Human Life Science, Osaka City University, Osaka, Japan; 9 Leading Program, Graduate School of Biomedical Sciences, Nagasaki University, Nagasaki, Japan

## Abstract

**Background:**

Tibetan highlanders have adapted to hypoxic environments through the development of unique mechanisms that suppress an increase in hemoglobin (Hb) concentration even in high-altitude areas. Hb concentrations generally decrease with increasing age. However, in the highlands, chronic altitude sickness is known to occur in the elderly population. To investigate how aging in a hypoxic environment affects Hb levels in Tibetan highlanders, we focused on the Mustang people, who live above 3500 m. We tried to clarify the pure relationship between aging and Hb levels in a hypoxic environment.

**Results:**

We found that the Hb concentration increased with increasing age in females but not in males. Multivariate analysis showed that age, pulse pressure, the poverty index, and vascular diameter were strongly correlated with the Hb concentration.

**Conclusions:**

We found unique Hb dynamics among female Tibetan highlanders. As seen in these Hb dynamics, there may be sex-based differences in the adaptive mechanism in Tibetan highlanders.

## Background

There are more than 140 million people living at altitudes above 2500 m worldwide [1]. Exposure to a unique environmental stress, e.g., high altitude, provides us with a natural situation that elicits cultural and/or biological responses. Natural human colonization of high-altitude plateaus on two continents, the Andean plateau (South America) and the Tibetan plateau (Asia), has resulted in two different arterial oxygen content phenotypes in indigenous Andeans and Tibetans [2]. In particular, Andeans appear to have adapted to high altitudes by developing distinctive characteristics to offset hypoxic stress at high altitude, e.g., by increasing hemoglobin (Hb) concentrations in an altitude-dependent manner; however, this leads to an increased risk of developing cardiovascular disease [3, 4]. In contrast, in Tibetan highlanders, Hb concentrations are not elevated at altitudes below 4000 m, and even at very high altitudes (over 4500 m), Hb concentrations in the Tibetan population remain the same or are only slightly elevated above the sea-level mean. These observations suggest that high-altitude erythrocytosis (i.e., an increase in red cell mass, indicated by increased Hb concentrations) is not an adaptive response among Tibetan highlanders [2, 5]. Accordingly, Moore et al. [6] argued that Tibetans are better adapted to high altitudes than Andeans because adaptation is a time-dependent process, and Tibetans have inhabited high altitudes for 25,000 years or possibly even 50,000 years, while Andeans have inhabited high altitudes for only 10,000 years [7–10].

On the genetic side, mutation rates of EGLN1 involved in the degradation of hypoxia-inducible factor (HIF) and EPAS1 involved in the expression of HIF itself have been reported to be particularly high among highlanders in Tibet [11, 12]. For example, the allele frequency of the EPAS1 gene rs13419896 is derived allele (A): ancestral allele (G) = 0.30: 0.70 in East Asia, A: G = 0.20: 0.80 in South Asia, A: G = 0.24: 0.76 in Nepalese. However, it was A: G = 0.77: 0.23 for the Sherpa people who are Tibetan highlanders. A variant of the hypoxic adaptation gene, which is inferior in lowland people, is predominant in Tibetan highlanders, and this genotype is involved in the mechanism that suppresses the increase in hemoglobin concentration even in a hypoxic environment [13, 14]. In addition, GWAS genetic analysis of Mustang people has reported that EPAS1 variants are associated with low hemoglobin concentrations [15].

Even among Tibetan highlanders who have acquired such a hypoxic adaptation, a slight increase in Hb is seen as the altitude increases, and some people are classified as having polycythemia [16]. In the case of Tibetan highlanders, it has been reported that Hb concentrations may also increase due to an imbalance of the hypoxic adaptation mechanism resulting from aging and changes in eating habits [17]. However, Hb levels generally tend to decrease with increasing age [18]. Similarly, it is assumed that the ability of Tibetan highlanders to synthesize Hb decreases with increasing age.

**Fig. 1:**
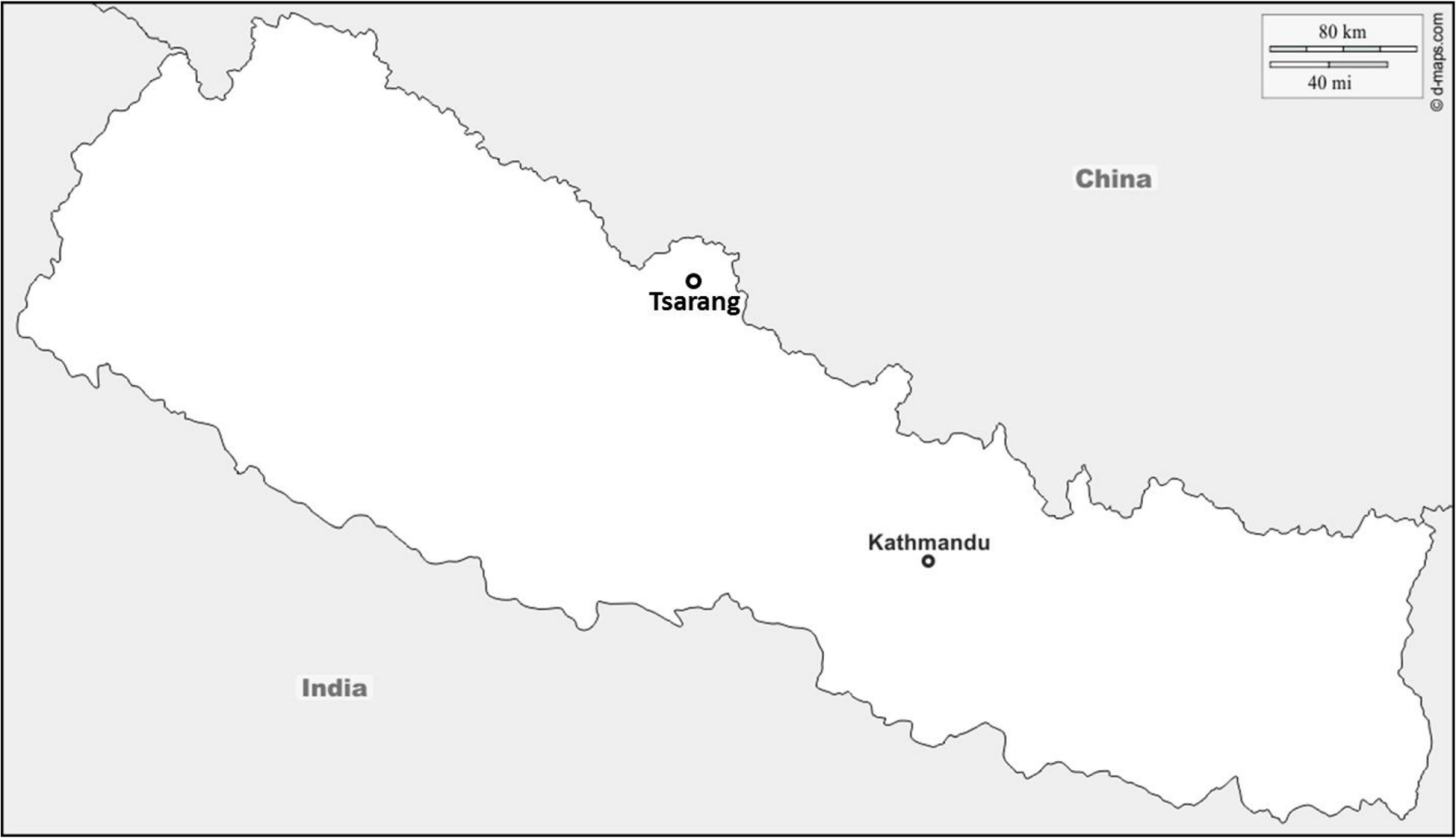
Location of Tsarang village. Tsarang is located in Mustang district in northern Nepal and is at an altitude of 3560 m

Tsarang village is located at an altitude of approximately 3560 m and contains a total of 452 people (132 households), accounting for 6.4% of the total population of the Mustang district [19, 20]. Tsarang village, Mustang district, Nepal, which is located adjacent to southern Tibet, used to be in the Mustang Kingdom (Fig. 1). It was founded in 1440 and continued to exist as an autonomous kingdom until 2008 [21, 22]. The Loba people living in Mustang share common ancestors with other Tibetans [22]. Moreover, the Mustang Kingdom restricted interactions with outside populations for a long period of time [5]. Therefore, by conducting a survey in Tsarang, where the population retains the genetic characteristics acquired by the Tibetan highlanders and has not yet been strongly influenced by modernization and changes in eating habits, we sought to clarify the pure relationship between aging and Hb levels in a hypoxic environment among Tibetan highlanders.

## Results

### Anthropometric and biochemical characteristics of participants by sex

In this study, we focused on Hb; the data from 76 males and 103 females as final objects were used. Hemoglobin was measured by near-infrared rays by inserting a finger into the measuring instrument, so that a person whose knuckle did not stretch could not insert the finger into the measuring instrument. In addition, people with strong discoloration of the finger skin could not be measured accurately by near infrared rays. As a result, these people were excluded because they could not measure hemoglobin concentration.

A summary of the medical examination results, which comprises the values of 10 items including age, body mass index (BMI), oxygen saturation (SpO_2_), systolic blood pressure (SBP), diastolic blood pressure (DBP), pulse pressure (PP), vascular diameter (VD), hemoglobin (Hb), glycated hemoglobin (HbA1c), and deprivation score (D score), is shown in Table 1. The age of the male participants was 44.66 ± 1.55 years, and the age of the female participants was 46.47 ± 1.66 years; thus, there was no age difference between the sexes (*p* = 0.4238). Additionally, BMI (23.00 (21.05–26.20) vs 23.10 (20.45–25.40), *p* = 0.5872), SpO_2_ (91.50 (90.00–93.00) vs 91.00 (89.50–93.00), *p* = 0.6062), HbA1c (5.80 (5.60–6.00) vs 5.85 (5.70–6.10), *p* = 0.0516), and D scores (0.22 (0.17–0.28) vs 0.22 (0.22–0.33), *p* = 0.2251) did not differ significantly between males and females.

**Table 1:** Anthropometric variables, biochemical data, and poverty index by sex. Values are presented as the mean ± SE or median (1st quartile–3rd quartile). Data were analyzed by Student’s *t* test or the Wilcoxon rank-sum test. The *D* score was considered poor at ≥ 33.3%

Sex	Male	Female	*p* value
*n* = 179	*n* = 76	*n* = 103	
Age	44.66 ± 1.55	46.47 ± 1.66	0.4238
BMI	23.00 (21.05–26.20)	23.10 (20.45–25.40)	0.5872
SpO_2_	91.50 (90.00–93.00)	91.00 (89.50–93.00)	0.6062
SBP	126.0 (116.4–139.5)	113.0 (106.8–126.2)	< 0.0001
DBP	78.50 (72.38–85.75)	74.50 (66.75–80.50)	0.0031
PP	47.50 (42.00–53.00)	42.00 (35.50–47.00)	0.0001
VD^a^	1.08 (0.83–1.26)	0.98 (0.74–1.13)	0.0148
Hb	14.50 (13.70–15.70)	13.10 (12.30–14.10)	< 0.0001
HbA1c^b^	5.80 (5.60–6.00)	5.85 (5.70–6.10)	0.0516
D score^c^	0.22 (0.17–0.28)	0.22 (0.22–0.33)	0.2251

^a^2 participants could not be measured due to severe deformation of finger joint or machine troubles

^b^14 participants could not be measured due to blood viscosity or machine troubles

^c^2 participants did not answer the question with questionnare

There were significant sex-based differences in SBP (126.0 (116.4–139.5) vs 113.0 (106.8–126.2), *p* < 0.0001), DBP (78.50 (72.38–85.75) vs 74.50 (66.75–80.50), *p* = 0.0031), PP (47.50 (42.00–53.00) vs 42.00 (35.50-47.00), *p* = 0.0001), VD (1.08 (0.83–1.26) vs 0.98 (0.74–1.13), *p* = 0.0148), and Hb (14.50 (13.70–15.70) vs 13.10 (12.30–14.10), *p* < 0.0001), and these values were higher in males than in females.

Variations in each variable were compared among the three age groups (18 to 40, 41 to 60, 61, or older) and are shown in Tables 2 and 3. In males, there were no significant differences in SpO_2_ (92.00 (91.00–93.00) vs 91.00 (88.75–92.25) vs 89.00 (87.50–90.50), *p* = 0.3695), SBP (123.5 (115.0–130.0) vs 133.0 (119.8–145.1) vs 117.5 (108.8–146.0), *p* = 0.5290), PP (47.50 (42.00–52.00) vs 48.75 (43.88–53.25) vs 45.50 (40.50–65.50), *p* = 0.3866), VD (1.00 ± 0.06 vs 1.09 ± 0.06 vs 1.03 ± 0.07, *p* = 0.4946), Hb (14.72 ± 0.25 vs 14.51 ± 0.24 vs 13.65 ± 0.39, *p* = 0.0960), HbA1c (5.60 (5.50–5.70) vs 5.90 (5.70–6.13) vs 6.00 (5.80–6.00), *p* = 0.4508) or *D* scores (0.28 ± 0.03 vs 0.23 ± 0.03 vs 0.22 ± 0.03, *p* = 0.2918) among the age groups. However, the values of BMI (22.67 ± 0.55 vs 24.60 ± 0.51 vs 21.74 ± 1.09, *p* = 0.0106) and DBP (68.68 ± 1.70 vs 83.30 ± 1.77 vs 76.27 ± 3.65, *p* = 0.0359) were significantly different among the age groups. Both were highest in the middle-aged group and relatively lower in the elderly group.

**Table 2:** Anthropometric variables, biochemical data, and poverty index by the age group in males. Values are presented as the mean ± SE or median (1st quartile–3rd quartile). Data were analyzed by ANOVA or the Kruskal-Wallis test to compare variables among the three age groups. The *D* score was considered poor at ≥ 33.3%

*n* = 76	Age group			*p* value
	18 to 40	41 to 60	61 or order	
	*n* = 33 (43.4%)	*n* = 32 (42.1%)	*n* = 11 (14.5%)	
BMI	22.67 ± 0.55	24.60 ± 0.51	21.74 ± 1.09	0.0106
SpO_2_	92.00 (91.00–93.00)	91.00 (88.75–92.25)	89.00 (87.50–90.50)	0.3695
SBP	123.5 (115.0–130.0)	133.0 (119.8–145.1)	117.5 (108.8–146.0)	0.529
DBP	68.68 ± 1.70	83.30 ± 1.77	76.27 ± 3.65	0.0359
PP	47.50 (42.00–52.00)	48.75 (43.88–53.25)	45.50 (40.50–65.50)	0.3866
VD^a^	1.00 ± 0.06	1.09 ± 0.06	1.03 ± 0.07	0.4946
Hb	14.72 ± 0.25	14.51 ± 0.24	13.65 ± 0.39	0.096
HbA1c^b^	5.60 (5.50–5.70)	5.90 (5.70–6.13)	6.00 (5.80–6.00)	0.4508
*D* score	0.28 ± 0.03	0.23 ± 0.03	0.22 ± 0.03	0.2918

^a^1 participant could not be measured due to severe deformation of finger joint or machine troubles

^b^7 participants could not be measured due to blood viscosity or machine troubles

**Table 3:** Anthropometric variables, biochemical data, and poverty index by the age group in females. Values are presented as the mean ± SE or median (1st quartile–3rd quartile). Data were analyzed by ANOVA or the Kruskal-Wallis test to compare variables among the three age groups. The *D* score was considered poor at ≥ 33.3%

*n* = 103	Age group			*p* value
	18 to 40	41 to 60	61 or order	
	*n* = 20 (19.4%)	*n* = 72 (69.9%)	*n* = 11 (10.7%)	
BMI	22.6 ± 0.56	24.18 ± 0.54	22.64 ± 0.68	0.0862
SpO_2_	93.00 (91.00–93.50)	91.00 (89.00–93.00)	88.00 (86.00–91.00)	0.1946
SBP	111.0 (105.8–117.0)	116.5.0 (107.5–133.0)	114.5 (107.8–131.5)	0.4754
DBP	74.21 ± 1.62	76.65 ± 2.11	72.87 ± 2.41	0.4342
PP	39.00 (34.00–43.25)	42.50 (36.50–48.00)	47.00 (38.75–53.00)	0.7192
VD^a^	0.93 ± 0.05	0.93 ± 0.04	0.96 ± 0.07	0.8640
Hb	12.61 ± 0.29	13.59 ± 0.25	14.05 ± 0.40	0.0034
HbA1c^b^	5.70 (5.58–5.80)	5.90 (5.80–6.10)	6.20 (6.00–6.30)	0.7647
*D* score^c^	0.26 ± 0.02	0.24 ± 0.02	0.27 ± 0.02	0.5547

^a^1 participant could not be measured due to severe deformation of the finger joint or machine troubles

^b^7 participants could not be measured due to blood viscosity or machine troubles

^c^2 participants did not answer the question with questionnare

In females, only Hb was significantly different among the three age groups and increased with increasing age (12.61 ± 0.29 vs 13.59 ± 0.25 vs 14.05 ± 0.40, *p* = 0.0034). BMI (22.6 ± 0.56 vs 24.18 ± 0.54 vs 22.64 ± 0.68, *p* = 0.0862), SpO_2_ (93.00 (91.00–93.50) vs 91.00 (89.00–93.00) vs 88.00 (86.00–91.00), *p* = 0.1946), SBP (111.0 (105.8–117.0) vs 116.5 (107.5–133.0) vs 114.5 (107.8–131.5), *p* = 0.4754), DBP (74.21 ± 1.62 vs 76.65 ± 2.11 vs 72.87 ± 2.41, *p* = 0.4342), PP (39.00 (34.00–43.25) vs 42.50 (36.50–48.00) vs 47.00 (38.75–53.00), *p* = 0.7192), VD (0.93 ± 0.05 vs 0.93 ± 0.04 vs 0.96 ± 0.07, *p* = 0.8640), HbA1c (5.70 (5.58–5.80) vs 5.90 (5.80–6.10) vs 6.20 (6.00–6.30), *p* = 0.7647), and *D* scores (0.26 ± 0.02 vs 0.24 ± 0.02 vs 0.27 ± 0.02, *p* = 0.5547) were not significantly different among the three age groups.

### Hb dynamics and related variables

**Fig. 2:**
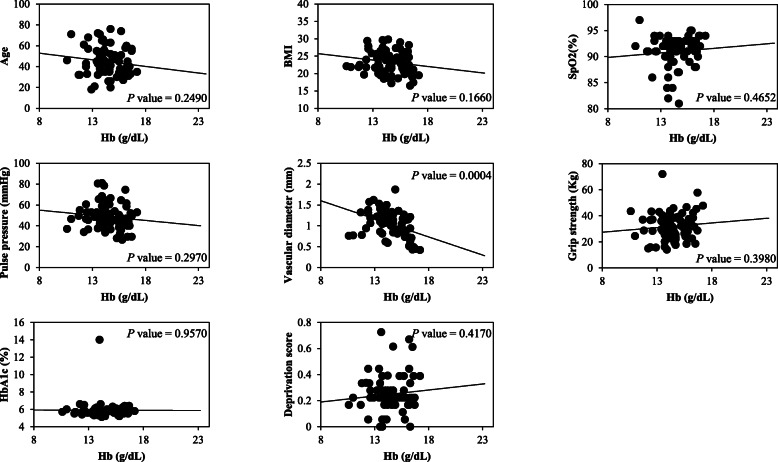
Linear regression analysis of Hb and each variable in males. Each variable was plotted against the Hb concentration in male participants. The *p* value indicates the result of linear regression analysis of Hb and each variable. Only vascular diameter was found to be significantly correlated with Hb

**Fig. 3:**
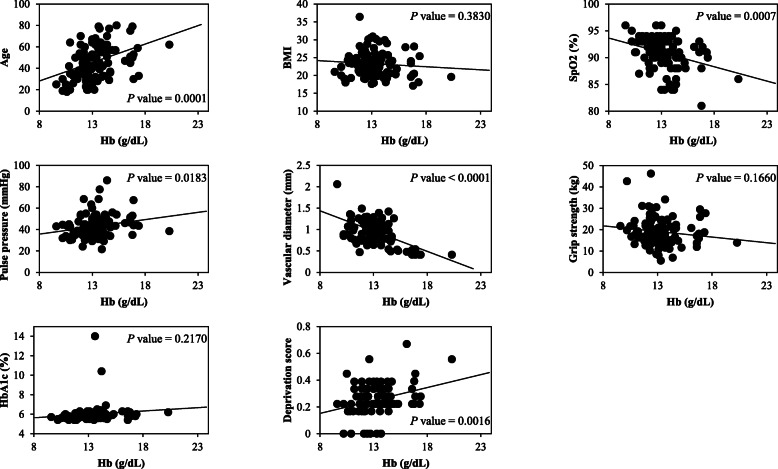
Linear regression analysis of Hb and each variable in females. Each variable is plotted against the Hb concentrations of female participants. The *p* values indicate the results of the linear regression analysis of Hb and each variable. Age, SpO_2_, pulse pressure, vascular diameter, and deprivation score were significantly correlated with Hb

The correlations between Hb and other variables were examined by linear regression analysis. The results for males are shown in Fig. 2, and the results for females are shown in Fig. 3. Only VD was associated with the Hb level in males (*p* = 0.0004). Age (*p* = 0.2490), BMI (*p* = 0.1660), SpO_2_ (*p* = 0.4652), PP (*p* = 0.2970), and grip strength: grip (*p* = 0.3980), HbA1c (*p* = 0.9570), and *D* score (*p* = 0.4170) were not associated with Hb.

In females, age (*p* = 0.0001), PP (*p* = 0.0183), and the *D* score (*p* = 0.0016) were positively correlated with Hb, and SpO_2_ (*p* = 0.0007) and VD (*p* < 0.0001) were negatively correlated. The remaining factors of BMI (*p* = 0.3830), grip (*p* = 0.1660), and HbA1c (*p* = 0.2170) were not correlated with the Hb value.

Based on these results, a multivariate analysis was performed to detect the variables correlated with the Hb value that were not influenced by confounding factors (Table 4). SBP and DBP were excluded as explanatory variables, and only PP maintained an association with blood pressure. In males, only VD was correlated with the Hb value (*p* = 0.0025), but in females, age (*p* = 0.0465), PP (*p* = 0.0438), *D* score (*p* = 0.0032), and VD (*p* < 0.0001) were strongly correlated with the Hb value.

**Table 4:** Results of the multiple regression analysis with Hb as the objective variable. Only vascular diameter was found to be significantly correlated with the Hb value in males. In females, age, pulse pressure, vascular diameter, and deprivation score were strongly correlated with the Hb value

Variables	Male				Female			
	Estimate	Std. error	*t* value	Pr(>|*t*|)	Estimate	Std. error	*t* value	Pr(>|*t*|)
Intercept	− 0.022119	0.120431	− 0.184	0.8549	0.013247	0.073509	0.18	0.8574
Age	− 0.176713	0.147861	− 1.195	0.2368	0.239542	0.118526	2.021	0.0465
BMI	− 0.030468	0.147861	− 0.22	0.8270	− 0.068577	0.079534	− 0.862	0.3910
SpO_2_	0.005151	0.143904	0.036	0.9716	− 0.109567	0.095932	− 1.142	0.2567
PP	− 0.035679	0.131199	− 0.272	0.7866	0.164115	0.080165	2.047	0.0438
HbA1c	0.067793	0.128064	0.529	0.5985	0.025044	0.08176	0.306	0.7601
Grip	0.055435	0.124306	0.446	0.6573	− 0.008568	0.083129	− 0.103	0.9182
D score	0.015942	0.123832	0.129	0.8980	0.229531	0.07571	3.032	0.0032
VD	− 0.401727	0.127334	− 3.155	0.0025	− 0.551756	0.074537	− 7.402	< 0.0001

In addition to Hb, SpO_2_ is also an indicator of blood oxygen dynamics. The relationship between SpO_2_ and aging by sex is shown in a scatter diagram (Fig. 3). SpO_2_ in both males and females showed a negative correlation with age (*p* = 0.0003, *p* < 0.0001).

## Discussion

### Physiological characteristics of highland residents

Table 1 shows the summaries of each variable and the sex-based differences in the health checkup results. Regarding the differences between sexes, the blood pressure, VD, and Hb values were higher in males than in females. Androgen, a male sex hormone, increases blood pressure through the renin-angiotensin system. On the other hand, estrogen, which is generally secreted in premenopausal females, has a dilating effect on blood vessels and decreases blood pressure. For these reasons, males generally have higher blood pressure than females in most age groups [23]. Furthermore, males have higher oxygen demands in tissues than females due to their greater muscle mass, so the blood vessel diameter in males is larger than that in females [24, 25]. It is known that these estrogen-induced vasodilatory effects and the difference in tissue oxygen demand between males and females cause sex-based differences in Hb production [26]. Therefore, it is believed that the sex-based differences in blood pressure, VD, and Hb values observed in this population were simply general sex-based differences that also occur in people living in lowlands [27, 28].

The BMI of middle-aged males was higher than that of younger and elderly people (Table 2); however, this tendency has also been reported in males living in lowlands [29]. Reduced metabolism is more likely to occur in middle-aged groups than in younger groups, and it can cause obesity. Moreover, dietary nutrient intake decreases with increasing age. It is suspected that these causes led to this result. DBP, like BMI, increases with increasing age but is known to decline after peaking between the ages of 50 and 60 years [30]. Previous studies have shown that elevated SBP, DBP, and PP values in young to middle-aged individuals indicate increased peripheral vascular resistance, but reduced DBP and elevated SBP and PP in middle-aged and elderly individuals indicate reduced aortic extensibility [31]. Aortic extensibility is a basic mechanical property associated with the elasticity of the vessel wall and is defined as a slight change in vessel cross-sectional area with respect to the pressure at which the vessel expands. This aortic extensibility is also a fundamental mechanical property that affects many vascular parameters such as systolic blood pressure and pulse pressure. In addition, it is well known that the aortic extensibility is reduced by factor such as aging, atherosclerosis, and hypertension [32].. Results similar to those associated with normal aging were obtained. Therefore, obesity and hypertension were identified as health risks in middle-aged males in this study.

Table 3 shows the differences in each variable among the female age groups, and only Hb was significantly different among the three groups. Regarding Hb dynamics, we made two unique observations. First, Hb increased with increasing age. This shows an opposite trend from that observed in people living in lowlands [18]. Second, Tibetan highlanders adapted to hypoxia by suppressing Hb elevation, but 10.7% (*n* = 11) of the females in this study were classified as polycythemic. A survey of Tibetan highlanders who are suspected to be of mixed ancestry, with Han Chinese and Tibetan highlander ancestors, showed that the proportion of individuals with polycythemia was approximately 15.7 ~ 25.5% in both males and females [12, 33]. Although the prevalence of polycythemia in our study was lower than that, it is interesting that an increase in Hb with increasing age and the onset of polycythemia were observed in only females. This is the first report of such sex-based differences.

### Elevation of Hb due to the loss of adaptive traits

Suppression of hemoglobin elevation in Tibetan highlanders involves a high-frequency missense mutation in the EGLN1 gene, which encodes prolyl hydroxylase 2 (PHD2). PHD2 essentially causes the degradation of hypoxia-inducible factor (HIF) that is triggers a hypoxic response. EGLN1 mutations in Tibetan highlanders enhance this mechanism and suppress the increase in hemoglobin concentration even in a hypoxic environment [11]. In addition, Tibetan highlanders have higher levels of nitric oxide (NO) in their blood than people living in lowlands [5, 6]. This enhances vasodilation and contributes to the maintenance of the oxygen cycle in the body in a hypoxic environment. This vasodilatory ability involves a mutation in the EPAS1 gene, which is said to have been inherited from Denisovans. In addition, the mutation of the EPAS1 gene is said to have also an suppressive effect on Hb elevation, and in this way, this population adapted to hypoxia while avoiding polycythemia [34]. However, to date, the mechanism underlying sex-based differences in Hb dynamics due to aging has not been reported. Therefore, the factors that correlated with the Hb value were analyzed stratified by sex. Only VD was significantly correlated with the Hb value in males (Table 4). This result indicates an enhanced adaptive ability in Tibetan highlanders. Moreover, it is considered that the high vasodilatory ability to maintain Hb within the normal range and sustain sufficient oxygen circulation has been maintained as an adaptive trait among male Tsarang residents.

On the other hand, the Hb value in females was found to be correlated with age, VD, PP, and the D score, according to the results of the multivariate analysis. In postmenopausal females, the amount of estrogen, which is associated with vasodilatory ability, is reduced, and VD decreases with increasing age [26]. The smaller the VD is, the greater the load on the blood vessels. It is thought that this also increases the PP. According to the result of PP by age, as shown in Tables 2 and 3, the median value in females at age 61 years and older was higher than that in males. It has been reported that the renin-angiotensin system may promote oxidative stress and cause the production of vasoconstrictors and a reduction in vasodilatory ability due to NO [23]. This is related to the mechanism underlying the relatively higher blood pressure in males than in females, but it is thought that elderly females with reduced estrogen secretion are also affected by this mechanism, which impairs vasodilatory ability. In this way, females may lose the vasodilatory characteristics of Tibetan highlanders. To supply sufficient oxygen to tissues despite a narrow VD, it may be necessary to increase the Hb concentration, which is contrary to adaptation. Furthermore, links between the poverty level and the risks of hypertension and cardiovascular disease have been reported [35, 36]. In other words, poverty may promote arteriosclerosis.

Hb and SpO_2_ are both indexes of blood oxygen circulation_._ SpO_2_, like Hb, usually decreases with increasing age [37]. Figure 3 shows the relationship between SpO_2_ and age in Tsarang residents stratified by sex. Among the residents of Tsarang, SpO_2_ which is greatly involved in the oxygen cycle of the living body, was also decreased with age in both men and women. From this result, it is possible that the biological stress that only women have to raise Hb is not the attenuation of oxygen binding ability but the vascular load.

For these reasons, age, PP, the D score, and VD may be involved in Hb elevation. In females who are vulnerable to vasodilation, the mechanism by which Hb elevation is suppressed, as observed in Tibetan highlanders, may not be necessary. In females who lose this adaptive trait, the Hb level increases when blood vessels narrow with increasing age, and some females may develop polycythemia. Additional studies with cross-altitude and cross-population analyses are necessary in the future.

The first limitation of this study is that we did not assess the plasma volume of Tibetan highlanders. The Tibetan highlanders are said to have acquired hypoxic adaptations that do not increase hemoglobin concentration. However, new findings was reported that Sherpa people increase both hemoglobin mass and plasma volume. In other words, it was suggested that the hemoglobin concentration of Tibetan highlanders is similar to that of lowlanders, but the mass of hemoglobin may actually increase [38]. Plasma has not received much attention in previous studies of hypoxic adaptation in Tibetan highlanders, and this study also focused on hemoglobin concentration and analyzed it. In the future, it is necessary to clarify more detailed biological reactions by investigating and analyzing the plasma volume, blood volume, and dehydration state. Furthermore, although this study did not investigate the association with sex hormone kinetics, we believe that women’s childbirth and regular menstruation in a hypoxic environment may be a significant burden on their bodies. In the future, it is necessary to clarify the relationship between sex hormones and the sex difference in hemoglobin dynamics in Tibetan highlanders.

## Conclusions

We identified new hemoglobin dynamics. The Hb level in female inhabitants of Tsarang did not decrease but rather increased with increasing age. As seen in these Hb dynamics, there may be sex-based differences in the optimal adaptive mechanism in Tibetan highlanders with regard to their hypoxic environment.

## Methods

### Subjects

We established a health camp at the health post in Tsarang village and conducted health checkups for highland residents in 2017. We asked the village mayor to disseminate information about the survey and posted a poster in the village to encourage them to participate in the survey. Of the participants, those who were under 18 years old, were pregnant, had a serious illness such as cancer, did not agree to participate, and were not born in the highlands were excluded from the final analysis.

### Setting variables

Regarding anthropometric measurements, Hb (ASTRIM FIT health monitoring analyzer: Sysmex, Kobe, Japan) and SpO_2_ (pulse oximeter: Masimo Radical V 5.0, Masimo Corp, CA, USA) were measured percutaneously. We classified participants as non-polycythemia (Hb < 18 g/dl for males and Hb < 16 g/dl for females) and polycythemia (Hb ≧ 18 g/dl for males and ≧ 16 g/dl for females) [17]. The Hb measuring instrument ASTRIM is equipped with a multi-wavelength light source from red to near infrared and a camera. Luminance information proportional to the amount of hemoglobin absorbed was obtained from the spectroscopic image of the peripheral blood vessel, and the blood vessel diameter was directly measured by the image. Height, weight, blood pressure (OMRON Model, HEM-7210, Kyoto, Japan), and grip strength were also measured. Glycated hemoglobin (HbA1c) in collected blood was measured by a Siemens DCA Vantage analyzer (Siemens Healthcare Diagnostics, Munich, Germany). Age, body mass index (BMI), SpO_2_ (%), systolic blood pressure (SBP, mmHg), diastolic blood pressure (DBP, mmHg), grip strength (grip, kg), Hb (g/dL), HbA1c (%), and vascular diameter (VD, mm) were the variables used for analysis. Pulse pressure (PP, mmHg) was calculated as the difference between the SBP and the DBP. Increased pulse pressure, which is systolic blood pressure minus diastolic blood pressure, is recognized as a risk of arteriosclerosis and cardiovascular disease [39]. The multidimensional poverty index (MPI) developed by the United Nations Development Program was adopted to evaluate the poverty level of residents. The deprivation score (*D* score), representing the poverty level of individuals, was calculated using the three dimensions of education, health, and standard of living. We used *D* score as poverty index of individuals in this study. Poverty was defined as a *D* score of 0.33 or higher [2]. Education includes data related to years of schooling and school attendance. Health includes data related to nutrition and child mortality. Standards of living include data related to cooking fuel, sanitation, drinking water, electricity, housing, and assets. These attribute data were obtained by questionnaire.

### Statistical analysis

**Fig. 4:**
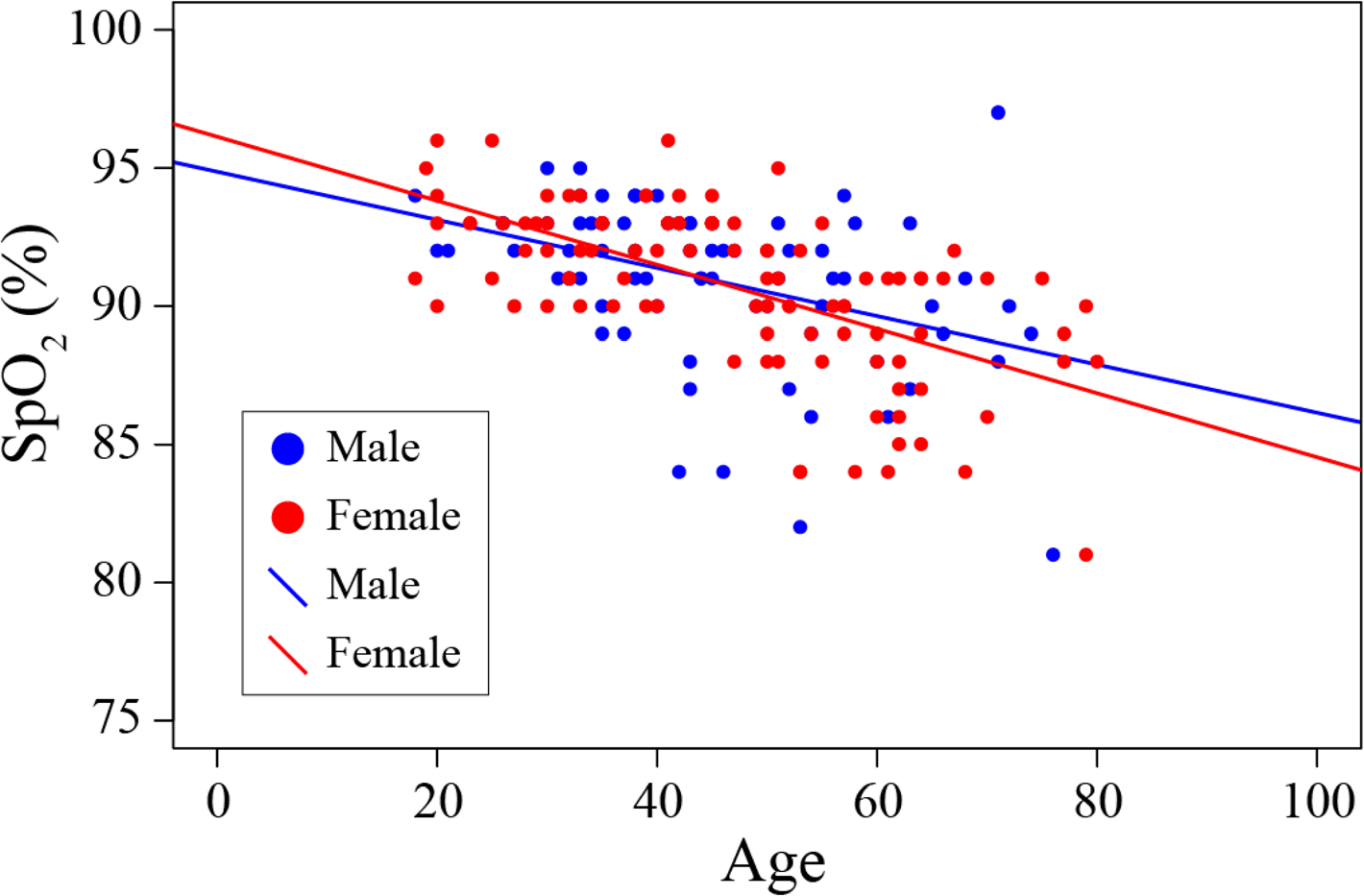
Relationship between SpO_2_ and age in each sex. Age plotted against SpO_2_ in participants. Males and females are indicated by blue and red circles, respectively. There was a negative correlation between age and SpO_2_ in each sex, and the approximate expressions for males and females were derived as *y* = − 0.0872*x* + 94.868 (*r* = − 0.4034193, *p* = 0.0003021, blue line) and *y* = − 0.1147*x* + 96.136 (*r* = − 0.6118297, *p* = 6.615e−12, red line), respectively, using Pearson correlation analysis

Medical examination data are presented as the means ± SEs or the medians (1st quartile–3rd quartile) (Table 1). We examined whether there were sex-based differences in each variable by performing Spearman’s *t* test or Wilcoxon’s rank-sum test on medical examination data. Next, to observe the effect of aging on each variable, the participants were divided into three age groups: younger (18 to 40), middle-aged (41 to 60), and older (61 or older). Significant differences in each variable among these three groups were tested by ANOVA or the Kruskal-Wallis test (Tables 2 and 3). Additionally, a univariate analysis with linear regression was performed (Figs. 2 and 3), followed by a multivariate analysis with multiple regression (Table 4) to identify factors correlated with the Hb value. For SpO_2_, which together with Hb reflects the state of oxygen circulation, the relationship with aging was plotted by sex, and Pearson correlation analysis was performed (Fig. 4). A *p* value < 0.05 was considered statistically significant. The software used for each analysis was R (ver. 3.5.3) and R studio.

## Data Availability

All data used in this study are not publicly available due to their planned use in future studies, but scientifically appropriate requests will be met after consideration by ethics committees.

## Abbreviations

ANOVA: Analysis of variance
BMI: Body mass index
D score: Deprivation score
DBP: Diastolic blood pressure
EPAS1: Endothelial PAS domain protein 1
HbA1c: Glycated hemoglobin
Grip: Grip strength
Hb: Hemoglobin
MPI: Multidimensional poverty index
SpO_2_: Oxygen saturation of hemoglobin
PTSD: Posttraumatic stress disorder
PP: Pulse pressure
SE: Standard error
SBP: Systolic blood pressure
VD: Vascular diameter
